# Molecular regulation of the expression of leptin by hypoxia in human coronary artery smooth muscle cells

**DOI:** 10.1186/s12929-014-0109-8

**Published:** 2015-01-09

**Authors:** Chiung-Zuan Chiu, Bao-Wei Wang, Kou-Gi Shyu

**Affiliations:** School of Medicine, College of Medicine, Fu-Jen Catholic University, New Taipei, Taiwan; Division of Cardiology, Shin-Kong Wu Ho-Su Memorial Hospital, 95 Wen- Chang Road, Taipei, Taiwan; Graduate Institute of Clinical Medicine, College of Medicine, Taipei Medical, University, Taipei, Taiwan

**Keywords:** Leptin, Human coronary artery smooth muscle cell, Hypoxia, Angiotensin II, c-Jun N-terminal kinase pathway

## Abstract

**Background:**

Leptin, produced mainly by white adipose tissue, is a hormone that promotes vascular smooth muscle cell (VSMC) migration and proliferation, a process involved in the pathophysiology of atherosclerosis. Leptin expression in human coronary artery smooth cell (HCASMC) is induced by hypoxia. However, our understanding of the process of atherosclerosis in HCASMC is only emerging. Since the mechanisms by which hypoxia regulates leptin in HCASMC are as yet unknown, this study aims to investigate the mechanics of molecular regulation of leptin expression in HCASMC under hypoxia. We subjected cultured HCASMCs to hypoxia for varying periods of time. Through use of different signal pathway inhibitors, we were able to sort out and identify the pathway through which hypoxia-induced leptin expression occurs.

**Results:**

Leptin mRNA and protein levels increased after 2.5% hypoxia for 2-to-4 hours, with earlier expression of angiotensin II (AngII) and reactive oxygen species (ROS). The addition before hypoxia of the c-Jun N-terminal kinase (JNK) pathway inhibitor (SP600125), JNK small interfering RNA (siRNA), AngII receptor blockers (ARBs; losartan), or N-acetyl-L-cysteine (NAC, an ROS scavenger), had the effect of inhibiting JNK phosphorylation and leptin expression. Gel shift assay and luciferase promoter study showed that leptin/activator protein 1 (AP-1) binding and transcriptional activity to the leptin promoter increased after hypoxia, and SP600125, JNK siRNA, losartan, and NAC abolished the binding and transcriptional activity induced by hypoxia. The use of SP600125, JNK siRNA, losartan, and NAC effectively inhibited the binding and transcriptional activity induced by hypoxia. Migration and proliferation, ROS generation, and the presence of leptin in the nuclei of HCASMCs also increased under hypoxia.

**Conclusion:**

Hypoxia in HCASMCs increases leptin expression through the induction of AngII, ROS, and the JNK pathway to enhance atherosclerosis in HCASMCs.

**Electronic supplementary material:**

The online version of this article (doi:10.1186/s12929-014-0109-8) contains supplementary material, which is available to authorized users.

## Background

Leptin is a hormone that was discovered in 1994, the product of research that has brought significant advances in the understanding of cardiovascular diseases (CVDs), obesity, and the metabolic syndrome [1]. Apart from its function as a regulator of energy expenditure, leptin also serves other roles, among them, leptin functions as a producer of a number of important chemical messengers [2]. The primary functions of leptin include appetite and energy balance, angiogenesis, insulin secretion, blood pressure control, vascular hemostasis, inflammation and immune response, and metabolic regulation [3-9]. Its production improves endothelial function, promotes angiogenesis, and reduces hypertension, atherosclerosis and inflammation. Some of Leptin’s peripheral effects, however, are negative, including the stimulation of vascular inflammation, oxidative stress, and vascular smooth muscle cell (VSMC) hypertrophy [3-16]. Several studies have shown an independent relationship between high leptin levels and atherosclerosis, myocardial infarction, stroke, and coronary artery intimal hyperplasia, suggesting that leptin may increase risks of CVDs [17,18].

Leptin is a hypoxia-inducible hormone. Its expression in increased by hypoxic conditions in various tissues such as placenta, pancreas, human skin dermal fibroblasts, adipocytes, and heart [4,19-22]. In fact, leptin is the gene most induced under hypoxia in human coronary arterial smooth muscle cell (HCASMC) [23]. Functional analyses of the leptin promoter reveal that hypoxic response elements mediate transcriptional activation by hypoxia. Furthermore, hypoxia in the adipocyte of white adipose tissue (a major site of leptin production) has been found to upregulate the secretion of a number of inflammation-related leptins [21]. Other studies have indicated that leptin is able to stimulate reactive oxygen species (ROS) generation in the cardiovascular system [24,25]. In addition, exogenous addition or stress-induced secretion of angiotensin II (AngII), a proatherogenic cytokine and a local mediator of inflammation, increases leptin synthesis in adipocytes and VSMCs [26,27].

Despite these findings, however, our understanding of the process of atherosclerosis in HCASMCs is only just emerging. To address the lack of detailed data in this field, this study aims to investigate the effect of hypoxia on leptin expression in HCASMCs. In particular, we will examine two related questions: whether hypoxia-induced pro-inflammatory cytokine (ex: AngII) secretion and ROS generation could trigger the induction of leptin, and whether this process is associated with the migration and proliferation of HCASMCs under hypoxia. This study will also investigate the signal pathways and molecular mechanisms mediating the expression of leptin by hypoxia in HCASMCs. Ultimately, a better understanding of the detailed mechanisms of leptin expression in hypoxic HCASMCs will provide us new insight in prevention and therapy for leptin-related atherosclerosis, a condition frequently encountered in patients suffering from CVDs.

## Methods

### Primary HCASMC culture

HCASMCs were obtained from Promo Cell GmbH (Heidelberg, Germany). The cells were cultured in growth medium supplemented with 10% fetal bovine serum, 100 U/ml penicillin, and 100 μg/ml streptomycin, all at 37°C in a humidified atmosphere of 5% CO2. Cells were grown to 90% confluence in 10 cm2 culture dishes and were sub-cultured in the ratio of 1:2. The enriched HCASMCs were then subjected to hypoxia.

### Hypoxia settings for HCASMC culture

Hypoxia was achieved by adding medium pre-equilibrated with nitrogen gas to the cells prior to incubation in a Plexiglas chamber purged with water-saturated nitrogen gas by an oxygen controller (PROOX model 110; BioSpherix, Ltd.; Redfield, NY). The partial pressure of oxygen (*p*O_2_) of the culture medium under hypoxia was monitored using an ISO_2_ dissolved oxygen meter (World Precision Instruments, Inc.; Sarasota, FL). Hypoxia culture media (BioSpherix C-chamber) was used with mixed air in and out controlled by a BioSpherix PROOX incubator. Hypoxia settings were as follows: 1) 10% O_2_, 5% CO_2_, and 85% N_2_; 2) 5% O_2_, 5% CO_2_, and 90% N_2_; 3) 2.5% O_2_, 5% CO_2_, and 92.5% N_2_. The use of a lower oxygen concentration (1%) was also tested, but the HCASMCs did not survive under such severe hypoxia. Hypoxia was applied for different hours. Researchers did not change the culture media throughout the experiment.

### Antibodies and reagents

We purchased recombinant leptin protein from PeproTech (Rocky Hill, NJ). Polyclonal antibodies against leptin and anti-AngII antibodies were obtained from Santa Cruz Biotechnology (Santa Cruz, CA). Polyclonal antibodies against c-Jun N-terminal kinase (JNK) and monoclonal antibodies (mAbs) against phospho-JNK were obtained from Cell Signaling (Beverly, MA, USA). PD98059, SB203580, and SP600125 were purchased from Calbiochem (San Diego, CA). Monoclonal anti-α-tubulin antibody and other reagents were obtained from Sigma (St. Louis, MO). The roles of JNK, p38 mitogen-activated protein kinase (MAPK), and extracellular signal-regulated kinase (ERK) in hypoxia-induced leptin expression were determined by pretreatment of the HCASMCs with 25 μM SP600125, 3 μM SB203580, or 10 μM PD98059 before hypoxia. SP600125 is a potent, cell-permeable, selective, and reversible inhibitor of JNK. SB203580 is a highly specific, cell-permeable inhibitor of p38 MAPK. PD98059 is a specific and potent inhibitor of the ERK pathway. To examine the effect of ARBs (AngII type 1 receptor blockers), HCASMCs were treated with 100 nM losartan (Merck & Co., Inc., Whitehouse Station, NJ). *N*-Acetyl-l-cysteine (NAC; a free radical scavenger), and Dp44mT (2,2′-dipyridyl-*N*,*N*-dimethylsemicarbazone; a ROS generator), and Anisomycin (a stimulator of JNK MAPK) were purchased from Calbiochem. In the respective experiments, cells were treated with 30 nM of Dp44mT, 500 μM of NAC, and 20 μM of Anisomycin.

### RNA isolation and reverse transcription

Total RNA was isolated from cells using a single-step acid guanidinium thiocyanate/phenol/chloroform extraction method. Total RNA (1 μg) was incubated with 200 U of Moloney-Murine Leukemia Virus reverse transcriptase in a buffer containing a final concentration of 50 mmol/L Tris-Cl (pH 8.3), 75 mmol/L KCl, 3 mmol/L MgCl_2_, 20 U of RNase inhibitor, 1 μmol/L poly-dT oligomer, and 0.5 mmol/L of each dNTP in a final volume of 20 μL. Primers were designed for detection of leptin gene expression (forward: 5′-GTGCCTTCCA- GTAGTATCTT-3′; reverse: 5′-AGCCACAAGAATCCGCACAGG-3′). The reaction mixture was incubated at 42°C for 1 hour and then at 94°C for 5 minutes to inactivate the enzyme. A total of 80 μL of diethyl pyrocarbonate treated water was added to the reaction mixture before storage at −70°C.

### Real-time quantitative PCR

A Lightcycler (Roche Diagnostics, Mannheim, Germany) was used for real-time PCR. cDNA was diluted to a ration of 1 in 10 with nuclease-free water. We used 2 μL of the solution for the Lightcycler SYBR-Green mastermix (Roche Diagnostics, Basel, Switzerland): 0.5 μmol/L primer, 5 mmol/L MgCl2, and 2 μL Master SYBR-Green in nuclease free water in a final volume of 20 μL. The initial denaturation phase for a specific gene was 5 min at 95°C followed by an amplification phase as detailed below: denaturation at 95°C for 10 sec; annealing at 63°C for 7 sec; elongation at 72°C for 8 sec; detection at 79°C and for 45 cycles. Amplification, fluorescence detection, and post-processing calculation were performed using the Lightcycler apparatus. Individual PCR products were analyzed for DNA sequence to confirm the purity of the product.

### Western blot analysis

After harvesting HCASMCs by scraping, we centrifuged (300 × g) the sample for 10 min at 4°C. The pellet was resuspended and homogenized in Lysis Buffer (Promega Corp., Madison, WI), centrifuging at 10,600 × g for 20 minutes. Bio-Rad Protein Assay was used to measure the protein content. Equal amounts of protein (15 μg) were loaded into a 12.5% sodium dodecyl sulfate (SDS)-polyacrylamide mini gel, followed by electrophoresis. Proteins were electroblotted onto nitrocellulose. The blots were incubated overnight in Tris-buffered saline containing 5% milk to block nonspecific binding of the antibody. Proteins of interest were revealed with specific antibodies as indicated (1:1000 dilution) for 1 hour at room temperature, followed by incubation with a 1:5000 dilution of horseradish peroxidase-conjugated polyclonal anti-rabbit antibody for 1 hour at room temperature. The membrane was then detected with an enhanced chemiluminescence detection system (Amersham, Buckinghamshire, England). Equal protein loading of the samples was further verified by staining mouse anti-tubulin mAbs. All Western blots were quantified using densitometry.

### RNA interference

HCASMCs were transfected with JNK-, leptin- and hypoxia inducible factor-1α (HIF-1α)-annealed small interfering RNA (siRNA; Dharmacon; Lafayette, CO). JNK1, leptin, or HIF-1α siRNAs are target-specific 19- to 21-nt siRNAs designed to knock down gene expression. The JNK1 siRNA sequences were 5′-CGUGGAUUUAUGGUCUGUGdTdT (sense) and 5′-CACAGACCAUAAAUCCACGdTdT (antisense). The leptin siRNA sequences were 5′-GCAGATAGCTCATGACCTGTT (sense) and 5′-AACAGGTCATGAGCTATCTGC (antisense). The HIF-1α siRNA sequences were 5′-UUACUGAGUUGAUGGGUUA (sense) and 5′-UAACCCAUCAACUCAGUAA (antisense). As a negative control, a control siRNA was used. HCASMCs were transfected with siRNA oligonucleotides, using Effectene transfection reagent according to the manufacturer’s instructions (Qiagen; Valencia, CA).

### Enzyme-linked immunosorbent assay (ELISA) for AngII and leptin

The level of AngII and leptin were measured by using a quantitative sandwich enzyme immunoassay (SPI-BIO; Massy, France), using a specific anti-AngII and anti-leptin antibodies, as previously described [28]. Conditioned medium from HCASMCs subjected to hypoxia and those from control cells were collected for AngII and leptin measurement. Both the intra-assay and the inter-assay coefficient of variance were less than 10%.

### Electrophoretic mobility shift assay (EMSA)

Nuclear protein concentrations from cells were determined by Bio-rad protein assay. Consensus and control oligonucleotides (Santa Cruz Biotechnology Inc., CA) were labeled by polynucleotides kinase incorporation of [γ-^32^P]. After radiolabeling the oligonucleotide, we mixed the nuclear extracts (4 μg of protein in 2 μl of nuclear extract) with 20 pmol of the appropriate [γ-^32^P]-labeled consensus or mutant oligonucleotide in a total volume of 20 μl for 30 minutes at room temperature. The consensus oligonucleotide sequence of activator protein 1 (AP-1) was 5′-CGCTTGATGACTCAGCCGGAA-3′. The AP-1 mutant oligonucleotide sequence was 5′-CGCTTGATGACTTGGCCGGAA-3′. The samples were then resolved on a 4% polyacrylamide gel. We used autoradiography to image dried and imaged gels. In each case controls were performed with mutant oligonucleotides or cold oligonucleotides to compete with labeled sequences.

### Luciferase reporter DNA construction, transient transfection, and luciferase assay

A human leptin promoter construct (−866/+35) with specific forward and reverse primers was generated and amplified as follows. The amplified product was digested with the restriction enzymes MluI and BglII and ligated into the pGL3-basic luciferase plasmid vector (Promega) digested with the same enzymes. The leptin promoter contains leptin-binding sites for AP-1 (CA) at position −596 to −595. We also changed the mutant sequence at position −596 to −595 from CA to TG. For the mutant, the leptin binding sites were mutated using a mutagenesis kit (Stratagene, La Jolla, CA). Site-specific mutations were confirmed by DNA sequencing. Plasmids were transfected into HCASMCs using a low pressure-accelerated gene gun (Bioware Technologies, Taipei, Taiwan) essentially following the protocol from the manufacturer. Test plasmid at 2 μg and control plasmid (pGL4-Renilla luciferase) 0.02 μg was co-transfected with gene gun in each well, and then replaced by normal culture medium. Following 2 hours of hypoxia, cell extracts were prepared using Dual-Luciferase Reporter Assay System (Promega) and measured for dual luciferase activity by luminometer (Glomax, Progega).

### Measurement of intracellular ROS

Intracellular reactive oxygen species (ROS) generation was measured using 5-(and-6)-chloromethyl-2′, 7′- dichlorofluorescein diacetate, acetyl ester (CM-H_2_DCFDA; Invitrogen, Carlsbad, CA), a cell-permeant indicator for ROS. We subjected confluent cells to serum starvation for 24 hours in preparation for experimentation. Starved cells were then treated with 2.5% hypoxia and NAC for 5 minutes at 37°C and then imaged by inverted fluorescence microscopy. ROS production was measured using the cell permeant probe 2′-7′-dichloro- dihydrofluorescin diacetate, which passively diffuses into cells in which intracellular esterase cleaves the acetate groups to form the impermeable 2′, 7′-dichlorofluorescin that remains trapped within the cell. After hypoxia treatment, we collected cells by trypsinization and resuspended them in phosphate-buffered saline medium. The ROS assay was performed according to the manufacturer’s instruction (Invitrogen, Eugene, OR). We used fluorescence microscopy to detect the green fluorescence.

### Migration assay

We determined the migration activity of HCASMCs using the growth factor-reduced Matrigel invasion system (Becton Dickinson, Franklin Lakes, NJ) following manufacturer’s protocol. We then seeded the Matrix gel (Chemicon International, Inc., Temecula, CA) with 5 × 10^4^ cells. Cells were then incubated at 37°C for 4 hours, with or without hypoxia. Three different phase-contrast microscopic high-power fields per well were photographed. The migratory cells with positive stain were counted by an observer blind to the experiment.

### Proliferation assay

We determined the proliferation of HCASMCs using [^3^H]Thymidine incorporation into the HCASMCs. Cells were seeded on ViewPlate (Packard Instrument, Meriden, CT) at a density of 5 × 10^3^ cells/well in a serum-free medium. Thymidine uptake was studied by addition of 500 nCi/ml [^3^H]Thymidine (Perkin Elmer, Boston, MA) for 2 to 4 hours, with or without hypoxia. Cells were washed twice with PBS. We studied nonspecific uptake in the presence of 10 μM cytochalasin B which was then subtracted from the measured value. MicroScint-20 (50 μl) was added, and the plate was read with Top Count (Packard Instrument).

### Statistical analysis

The data were expressed as mean ± SD. Statistical significance was performed with analysis of variance (GraphPad Software Inc., San Diego, CA). The Tukey-Kramer comparison test was used for pairwise comparisons between multiple groups after the ANOVA. We denoted statistical significance at a value of P < 0.05.

## Results

### Hypoxia increases the expression of leptin in cultured HCASMCs

**Figure 1:**
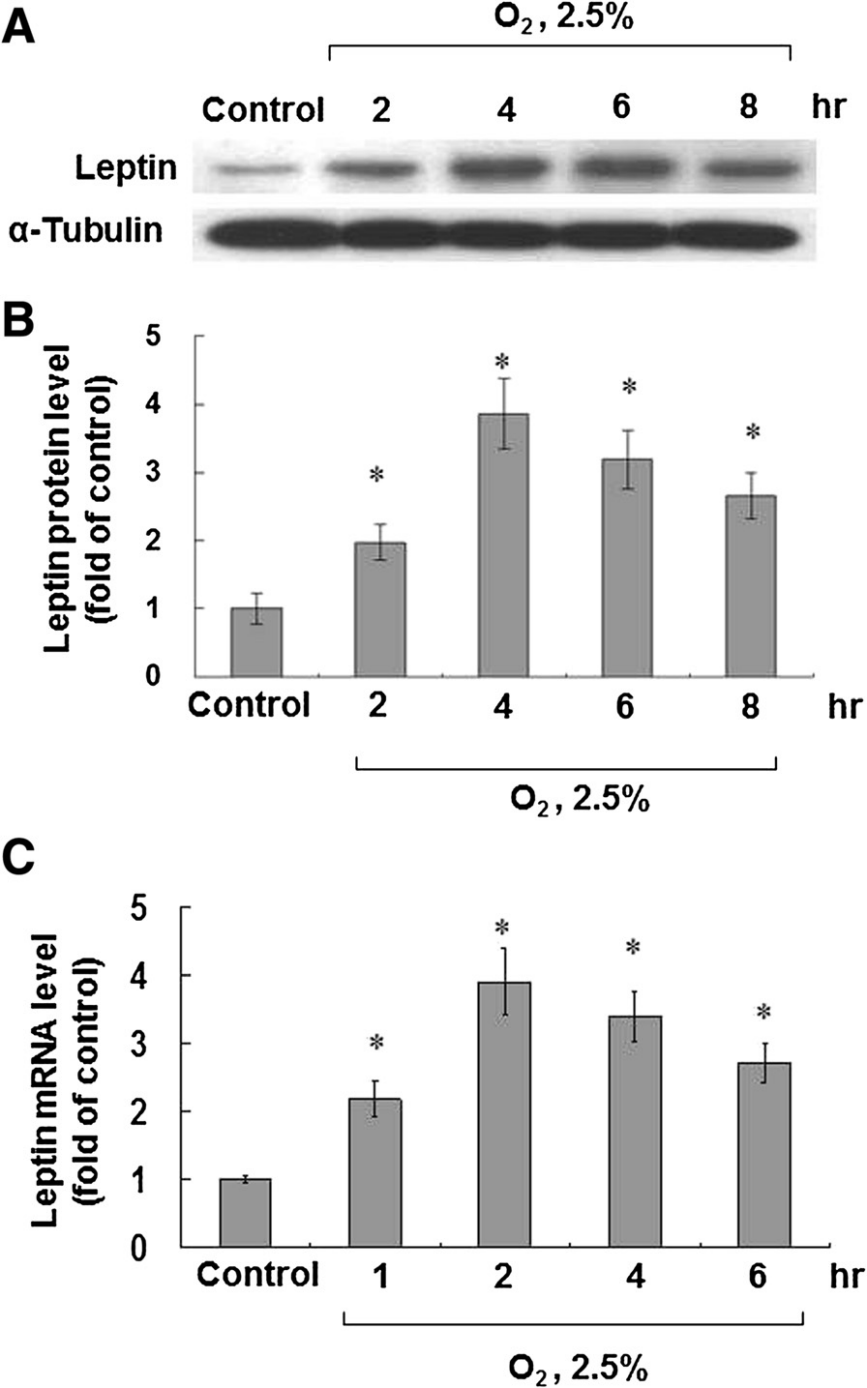
**Effect of 2.5% O**_**2**_ **hypoxia on leptin expression in human coronary artery smooth muscle cells (HCASMCs). (A and B)** Leptin protein levels increased, reaching a peak after 4 hours of 2.5% O_2_ hypoxia. We subjected HCASMCs to hypoxia for 2 to 8 hours. Total cell lysates were harvested and Western blot was performed using anti-leptin antibody. Alpha-tubulin was used as a protein loading control in each lane. ^*^P < 0.01 *vs.* normoxia control (n = 3). **(C)** Leptin mRNA level reached a peak after 2 h of 2.5% O_2_ hypoxia then declined. We used GAPDH as an internal control. ^*^P < 0.01 *vs.* normoxia control (n = 3).

We used different settings of hypoxia (10%, 5%, and 2.5%) for 4 hours as we laid out in the methods section (Additional file 1: Figure S1). We noted the greatest effect on the leptin protein expression at 2.5% hypoxia for 4 hours, as compared with 10% and 5% hypoxia. Therefore, we used 2.5% hypoxia for further analysis. Subjected to 2.5% hypoxia, the protein levels of leptin increased gradually and reached a peak after 4 hours (Figures 1A and B). The mRNA expression levels of leptin also reached their maximum after 2 hours and then decreased gradually (Figure 1C).

### Hypoxia increases the expression of leptin in cultured HCASMCs through the JNK pathway

**Figure 2:**
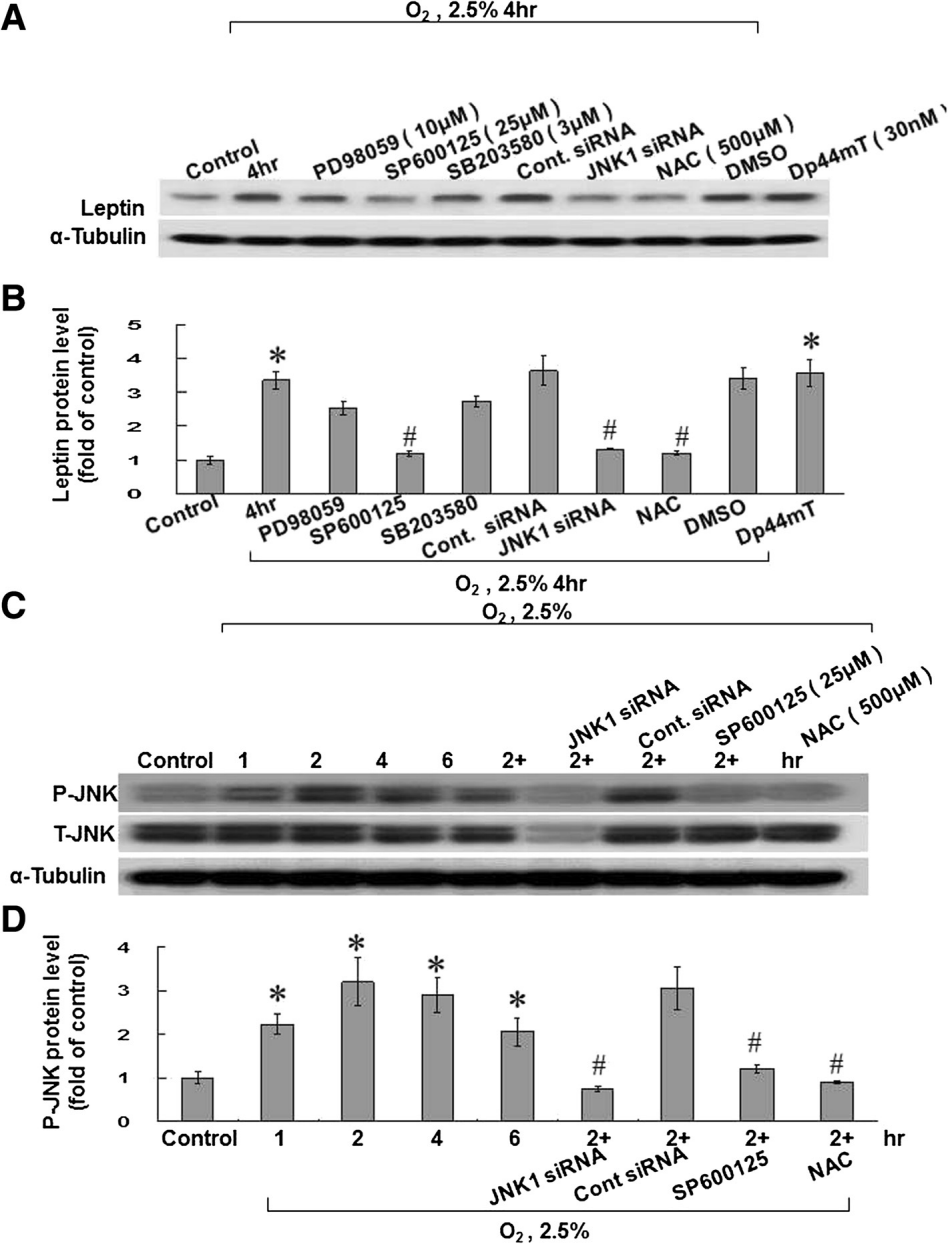
**The JNK pathway mediates hypoxia-induced leptin expression in HCASMCs. (A and B)** The c-Jun N-terminal kinase (JNK) inhibitor (SP600125), JNK small interfering RNA (siRNA), and N-acetyl-L-cysteine (NAC; a ROS scavenger) all blocked hypoxia-induced leptin expression. Exogenous addition of Dp44mT (an ROS generator; 30 nM)) under normoxia also increased leptin expression. We pretreated HCASMCs with an extracellular signal-regulated kinase (ERK) pathway inhibitor (PD98059; 50 μM), a JNK inhibitor (SP600125; 25 μM), a p38 mitogen-activated protein kinase (MAPK) inhibitor (SB203580; 3 μM), NAC (500 μM), or JNK1 siRNA prior to hypoxia for 4 hours. The HCASMCs were then harvested and analyzed by western blotting using an anti-leptin antibody. The results were normalized to α-tubulin. ^*^P < 0.01 *vs*. normoxia control. ^#^P < 0.01 *vs.* 4 h (n = 3). **(C and D)** Phosphorylation of the JNK mediated hypoxia-induced leptin expression in HCASMCs, which was blocked by SP600125, JNK1 siRNA, and NAC. HCASMCs were subjected to normoxia or hypoxia for different durations in the presence or absence of inhibitors. Cell lysates were collected for western blot analysis, using antibody for total and phospho-JNK. T-JNK = total JNK. P-JNK = JNK phosphorylation. ^*^P < 0.01 *vs*. normoxia control. ^#^P < 0.01 *vs.* 2 h (n = 3).

We used different signal pathway inhibitors to identify the signal transduction pathways of leptin under hypoxia (PD98059: ERK pathway inhibitor, SP600125: JNK inhibitor, and SB203580: P38 inhibitor). The JNK inhibitor (SP600125) produced the most immediately evident effect on Leptin expression, with the siRNA of JNK1 and NAC achieving the same effect (Figures 2A and B; Additional file 2: Figure S2). The solvent of the inhibitors (dimethyl sulfoxide; DSMO) did not inhibit hypoxia-induced expression of leptin. The exogenous addition of Dp44mT (an ROS generator) under normoxia also increased leptin expression (Figure 2A and B). We also found that JNK protein phosphorylation increased to its maximal level at 2 hours after exposure to 2.5% hypoxia, after which it gradually declined. SP600125 and JNK siRNA could effectively block phosphorylation of JNK protein. In addition, phosphorylation of the JNK protein could also be suppressed by NAC (Figures 2C and D).

### Hypoxia increases leptin levels with earlier AngII secretions in cultured HCASMCs

**Figure 3:**
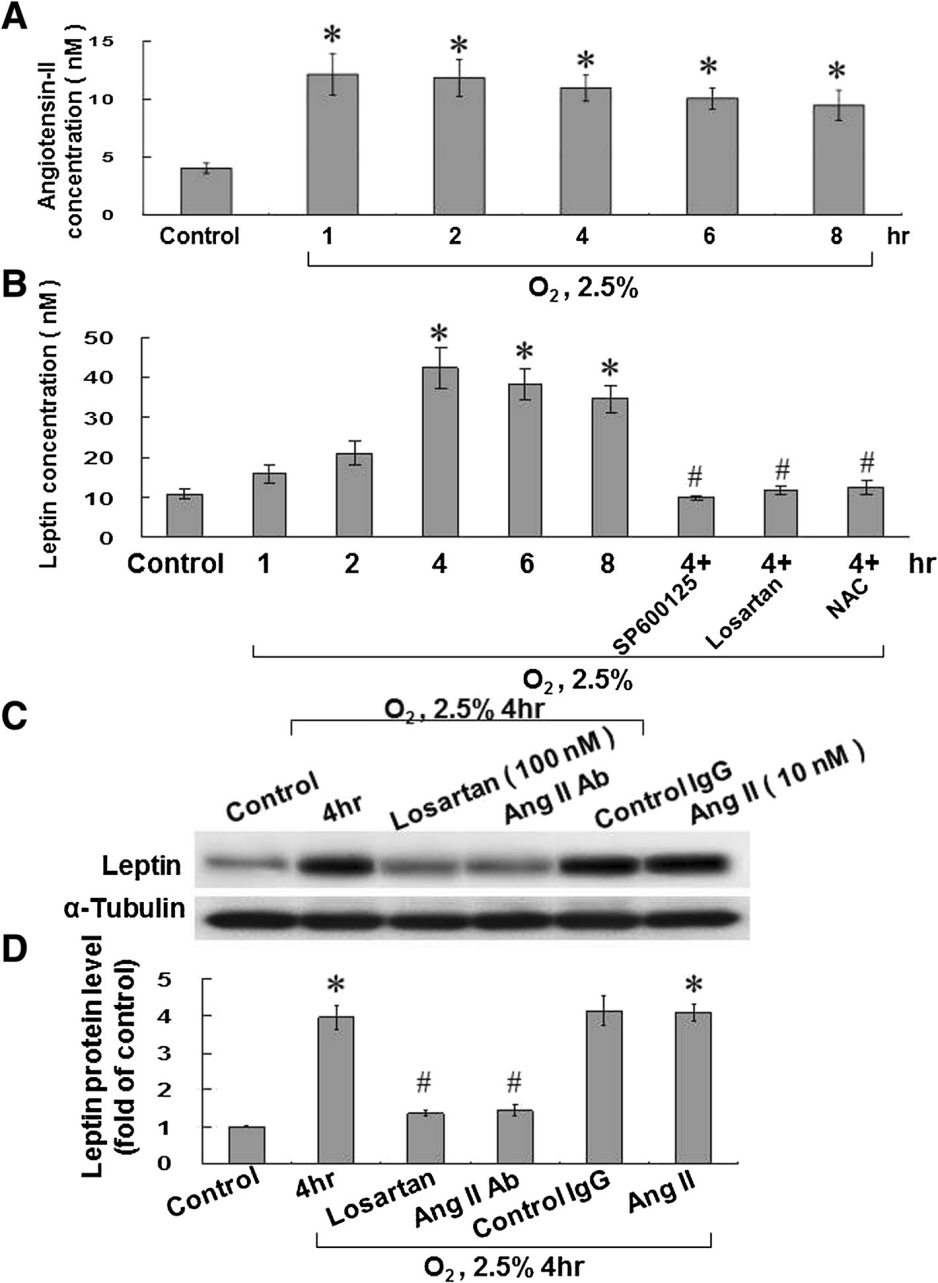
**Angiotensin II (AngII) mediates hypoxia-induced leptin expression in HCASMCs. (A and B)** AngII and leptin were measured in cell lysates and the culture medium by a quantitative, competitive Enzyme-linked immunosorbent assay (ELISA) using a specific anti-AngII and anti-leptin antibodies. *P < 0.01 *vs.* normoxia control. ^#^P < 0.01 *vs.* 4 h (n = 3). **(C and D)** Hypoxia-induced leptin expression was suppressed by AngII antibody and losartan. Exogenous addition of AngII (under normoxia) also increased leptin expression. HCASMCs were subjected to normoxia or hypoxia for 4 h and total cell lysates were immunoblotted with anti-leptin antibody. Alpha-tubulin was used as a protein loading control in each lane. *P < 0.01 *vs.* normoxia control. ^#^P < 0.01 *vs.* 4 h (n = 3).

Under 2.5% hypoxia, the AngII level increased and reached a peak after 1 hour, after which it began to decline. This timing of peak-and-decline happened considerably earlier than that of leptin levels, which reached their peak after 4 hours (Figures 3A and B). The addition of SP600125, losartan, and NAC before hypoxia significantly decreased leptin secretion from HCASMCs (Figure 3B). Exogenously added AngII (10 nM) also increased the leptin protein expression to a similar degree as 2.5% hypoxia (Figures 3C and D). The hypoxia-induced increase in the leptin protein level could be suppressed by ARB (losartan: 100 nM) and the AngII antibody (Figures 3C and D).

### Hypoxia induces intracellular ROS formation

**Figure 4:**
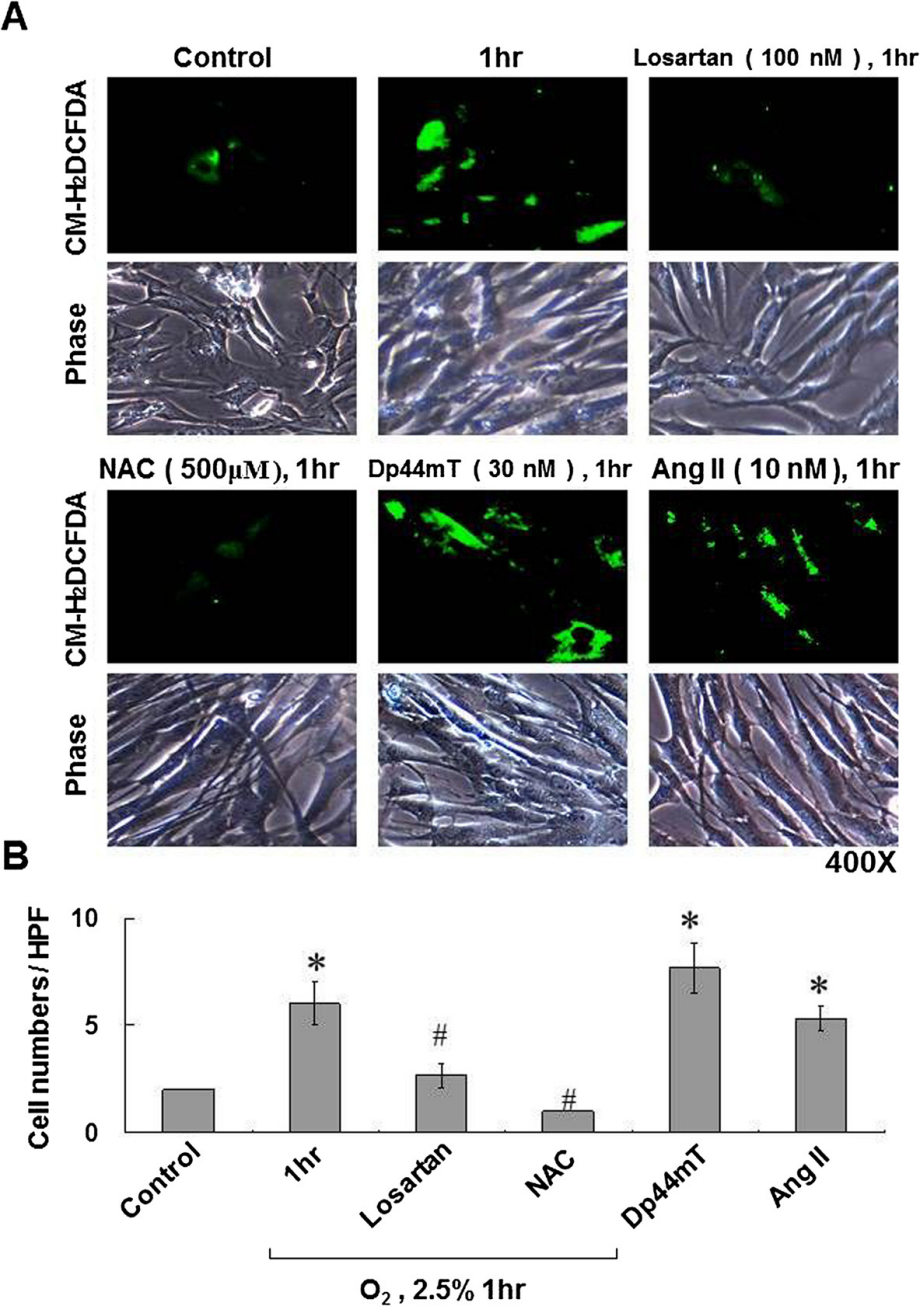
**Hypoxia-induced leptin expression in HCASMCs is mediated by reactive oxygen species (ROS). (A and B)** ROS assay, using a fluorescence microscope, indicated that hypoxia for 1 hour increased ROS production similarly to that of the exogenous addition of Dp44mT or AngII. Treatment with NAC (500 μM) or AngII type 1 receptor blocker (ARB; losartan 100 nM) 1 hour before hypoxia significantly blocked the induction. *P < 0.01 *vs.* normoxia control. ^#^P < 0.01 *vs.* 1 h (n = 3).

Intracellular ROS production increased after hypoxia for 1 hour (Figures 4A and B). Both Dp44mT (A ROS generator) and AngII achieved the same effects. NAC (an ROS scavenger) and losartan were noted to be effective in repressing hypoxia-induced intracellular ROS generation (Figure 4A and B).

### Hypoxia increases the binding between leptin and AP-1 and enhances transcriptional activity to the promoter of leptin in HCASMCs

**Figure 5:**
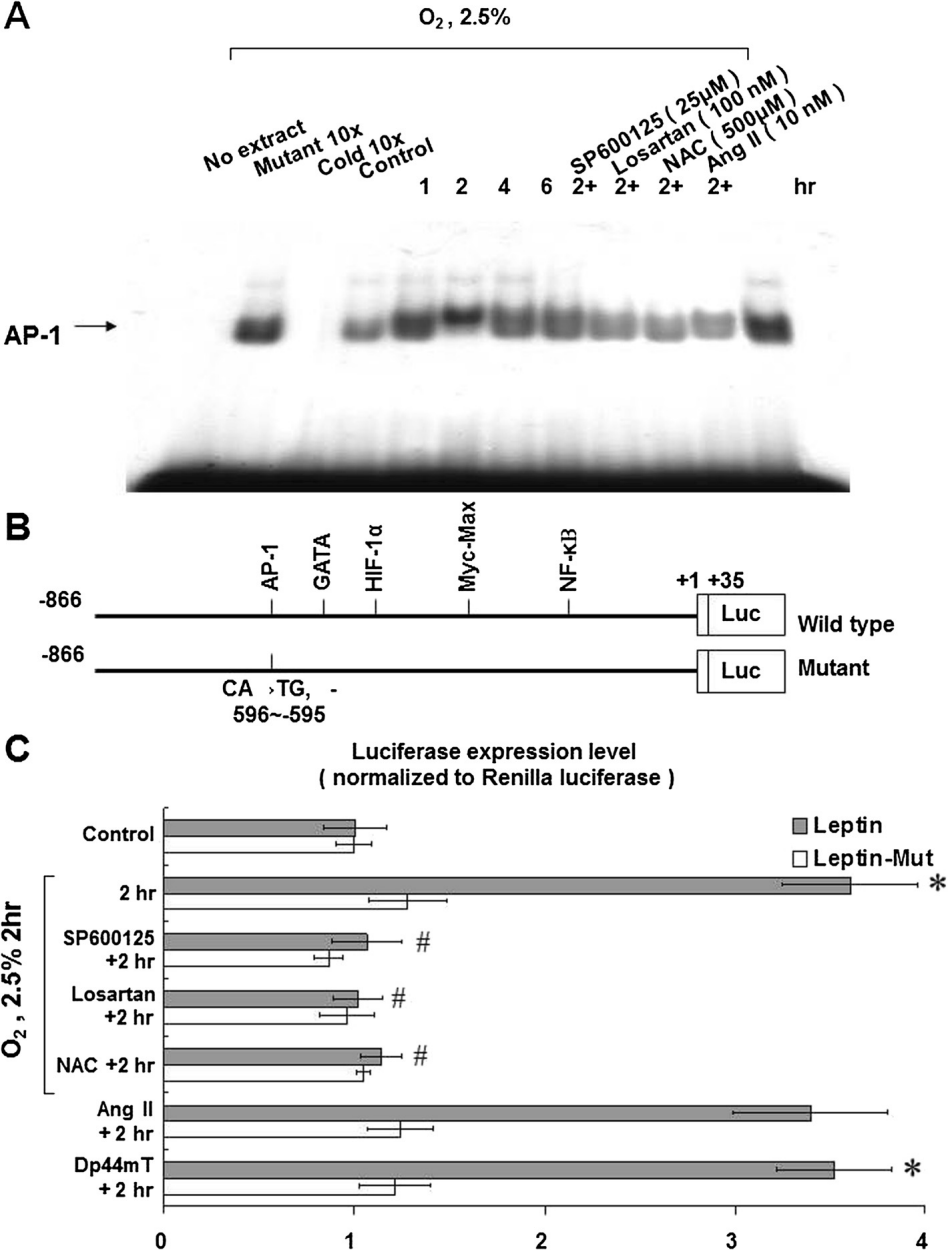
**Binding activity between leptin and activator protein 1 (AP-1) transcription factor and genetic transcription activity at the AP-1-binding site of the leptin promoter increase in HCASMCs under hypoxia. (A)** Electrophoretic mobility shift assay (EMSA) showed an increase in binding between leptin and AP-1 in HCASMCs under 2.5% O_2_ hypoxia. The binding between leptin and AP-1 was suppressed by SP600125 (25 μM), losartan (100 nM), and NAC (500 μM). **(B and C)** The luciferase reporter assay revealed that 2.5% O_2_ hypoxia increased the transcriptional activity of AP-I to the leptin promoter compared to the leptin mutant. Transcriptional activity was suppressed by SP600125, NAC, and losartan. Exogenous addition of AngII (10 nM) increased the transcriptional activity of HCASMCs similarly to that of 2.5% O_2_ hypoxia. *P < 0.01 *vs.* normoxia control. ^#^P < 0.01 *vs.* 2 h (n = 3).

Under 2.5% hypoxia, electrophoretic motility shift assays (EMSA) revealed increased binding activity between leptin and AP-1 (Figure 5A). This finding indicates that applying hypoxia to HCASMCs may increase the interaction between leptin and AP-1, as well as leptin expression and leptin/AP-1-binding activity. In addition, the binding between leptin and AP-1 could be effectively inhibited by SP600125, losartan, and NAC.

We also used a luciferase reporter assay to identify the genetic transcription activity of leptin in HCASMCs under hypoxia (Figures 5B and C). We found that hypoxia increased transcriptional activity of the leptin promoter. Leptin mutants failed to manifest the same effect as wild-type leptin under hypoxia. Exogenously added AngII also increased the transcriptional activity of leptin in HCASMCs. SP600125, NAC, and losartan suppressed the transcriptional activity of leptin to HCASMCs. Therefore, both exogenously added AngII and hypoxia-induced AngII secretion resulted in leptin/AP-1 complex-associated gene transcription in HCASMCs. We also found that hypoxia-induced leptin expression may be independent to HIF-1α in HCASMCs. Because hypoxia-induced leptin expression could not be inhibited by siRNA of HIF-1α in our experiment (Additional file 3: Figure S3).

### AngII, ROS, and the JNK pathway are involved in leptin expression in HCASMCs under hypoxia

**Figure 6:**
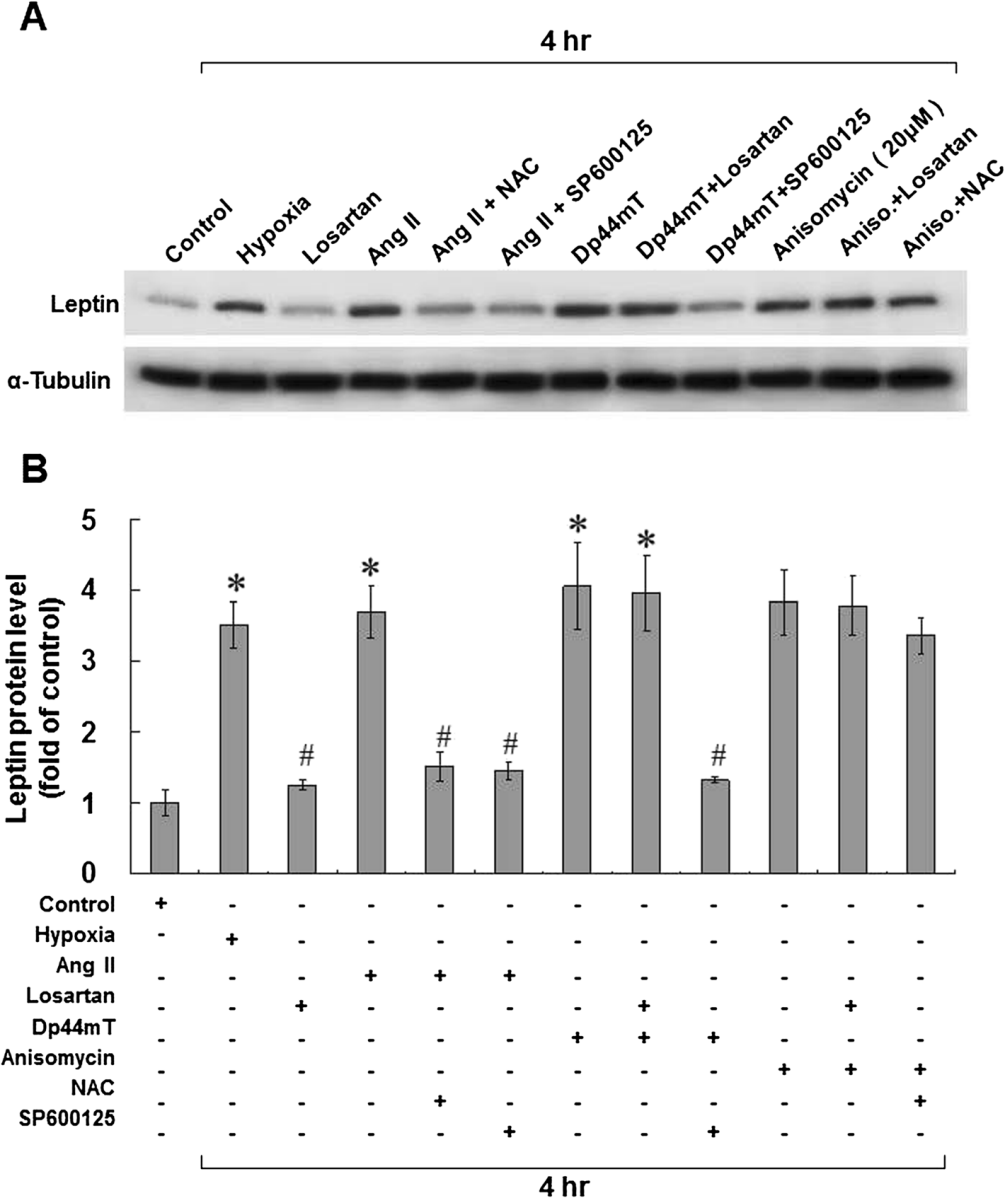
**AngII, ROS, and the JNK pathway are involved in leptin expression in HCASMCs under hypoxia. (A and B)** Under hypoxia, AngII (10 nM), an ROS generator (Dp44mT; 30 nM)), and a JNK MAPK stimulator (Anisomycin; 20 μM) increased leptin protein expression in HCASMCs. AngII-induced leptin protein expression could be inhibited by SP600125 (25 μM) and NAC (500 μM). Dp44mT-induced leptin protein expression could be inhibited by SP600125 (25 μM). However, Anisomycin-induced leptin protein expression could not be inhibited by losartan (ARB; 100 nM) or NAC (500 μM). These results imply that hypoxia-induced leptin expression in HCASMCs is regulated through the JNK pathway, with its earliest expression coming from treatment with AngII, and then from ROS. *P < 0.01 *vs.* normoxia control. ^#^P < 0.01 *vs.* 4 h (n = 3).

As shown in Figure 6, treatment with hypoxia, AngII, an ROS generator (Dp44mT), and a JNK MAPK stimulator (Anisomycin) increased leptin protein expression in HCASMCs. AngII-induced leptin protein expression could be inhibited by SP600125 and NAC. Dp44mT-induced leptin protein expression could be inhibited by SP600125. However, Anisomycin-induced leptin protein expression could not be inhibited by losartan (ARB) or NAC. These results imply that hypoxia-induced leptin expression in HCASMCs is regulated through the JNK pathway, with its earliest expression coming from treatment with AngII, and then from ROS.

### Hypoxia increases HCASMCs migration

**Figure 7:**
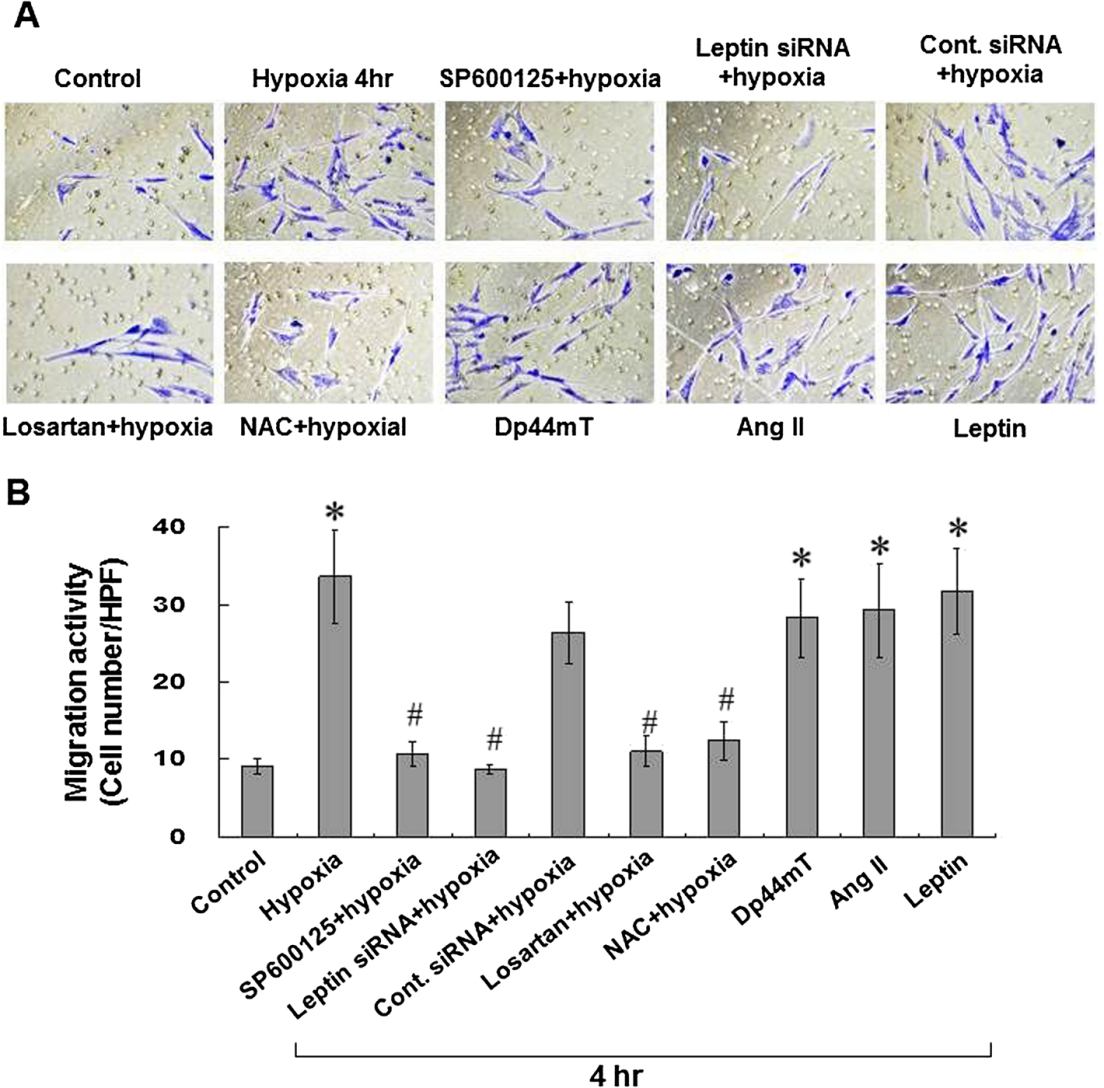
**Hypoxia increases migration activity of HCASMCs. (A and B)** 2.5% O_2_ hypoxia for 4 h increased migration activity of HCASMCs, which was inhibited by SP600125 (25 μM), leptin siRNA, NAC (500 μM), or losartan (100 nM). In addition exogenous addition of Dp44mT (30 nM), leptin, or AngII (10 nM) under normoxia also increased migration activity of HCASMCs. *P < 0.01 *vs.* normoxia control. ^#^P < 0.01 *vs.* 4 h (n = 3).

We examined migration activity in order to test the effect of hypoxia on the function of HCASMCs. As shown in Figure 7, treatment with hypoxia for 4 hours significantly increased the migration activity of HCASMCs. Pretreatment with SP600125, losartan, and leptin siRNA significantly blocked the induction of migration by hypoxia in HCASMCs. The control siRNA did not inhibit the migration activity induced by hypoxia. In addition, exogenous additions of AngII, Dp44mT, or leptin also significantly increased the migration activity of HCASMCs.

### Hypoxia increases human CASMCs proliferation

**Figure 8:**
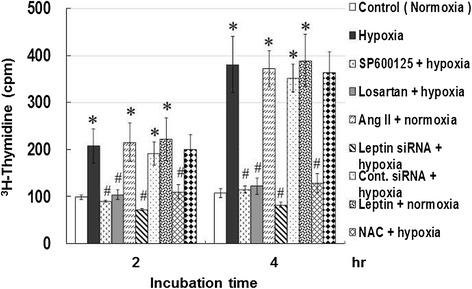
**Hypoxia increases proliferation activity of HCASMCs.** Incorporation of [^3^H]Thymidine into HCASMCs increased under 2.5% hypoxia and exogenous addition of leptin, AngII, or Dp44mT (under normoxia) for 2 to 4 h. Hypoxia-induced incorporation of [^3^H]Thymidine into HCASMCs was suppressed by leptin siRNA, losartan, NAC, and SP600125. *P < 0.01 *vs.*normoxia control. ^#^P < 0.01 *vs.* hypoxia (n = 6).

Hypoxia in HCASMCs increased cell proliferation after 2 to 4 hours; the increase was inhibited by SP600125, losartan, leptin siRNA, and NAC (Figures 8). In addition, exogenously added AngII or Dp44mT (an ROS generator) also increased [^3^H]-thymidine incorporation into HCASMCs after applying 2.5% hypoxia for 2 to 4 hours.

### Hypoxia increases the presence of leptin in the nuclei of HCASMCs

We used confocal microscopy to identify the presence of leptin in the nuclei of HCASMCs under hypoxia (Additional file 4: Figure S4). Hypoxia increased leptin expression (green color) in the nuclei (purple color) of HCASMCs (red color), which could be inhibited by SP600125, losartan, NAC, and leptin siRNA. In addition, the exogenous addition of AngII, Dp44mT, or leptin also increased the presence of leptin in the nuclei of HCASMCs.

## Discussion

Leptin plays an important role in atherosclerosis by initiating leukocyte and macrophage recruitment to the endothelial wall. With respect to CVDs, leptin also has a variety of pro-atherogenic functions. It stimulates the hypertrophy and proliferation of VSMCs and their production of metalloproteinase. In addition, leptin promotes the production of proliferative and inflammatory cytokines, it increases platelet aggregation and enhances the secretion of pro-atherogenic lipoprotein lipase by cultured human and rodent macrophages [16] causing endothelial dysfunction by increasing ROS [29]. Increased leptin level promotes VSMC proliferation and migration and vascular calcification. The generation of ROS by leptin reduces the bioavailability of nitric oxide (NO) in VSMCs by inactivating NO or eNOS and leads to endothelial dysfunction [30]. With respect to CVDs, leptin induces hypertension, atherosclerosis, myocardial infarction, vascular inflammation, VSMC hypertrophy and endothelial dysfunction. Thus, leptin induces phenotypic changes in VSMCs (proliferation, migration, hypertrophy, and calcification) that are pro-atherogenic.

Leptin also regulates fat metabolism and cardiovascular function. The expression of leptin is related to the development of hypoxia in the adipose tissue of obese individuals. Studies have found that white adipose tissue becomes hypoxic as size and mass expand due to obesity, the level of leptin is thus directly related to body weight or fat [31,32]. The development of hypoxia, furthermore, initiates and drives the inflammatory response and oxidative stress in the adipose tissue of obese individuals; this, again, is related to the expression of leptin [33]. The expression, under hypoxia, of the leptin gene linked to inflammation suggests a connection between hypoxia and the inflammatory response. It is well known, after all, that HIF-1α and leptin regulate the molecular response to hypoxia [34,35]. Under hypoxia, leptin has been found to be transcriptionally regulated by HIF-1α, with an increase of HIF-1α levels associated with the recruitment of leptin, which is a hypoxic transcription factor [36-38]. Moreover, the synthesis of leptin and vascular endothelial growth factor (VEGF) under hypoxia reflects the growth of the capillary network and the subsequent enhancement of blood delivery and tissue oxygenation to hypoxic areas that are typical responses to low O2. With this in mind, the inflammatory response incited by the increased production of leptin is associated with angiogenesis in adipose tissue [37-40]. In this light, hypoxia is understood as an initiating event in adipose tissue dysfunction, followed by fibrosis and inflammation. Also, obstructive sleep apnea with hypoxia in obese people is associated with insulin resistance, metabolic syndrome, and cardiovascular abnormalities [41,42]. In *in vivo* studies, intermittent hypoxia has been found to induce insulin resistance in mice, and this is accompanied by an up-regulation of leptin gene expression in adipose tissue [43].

In this study, we identified some findings not previously reported: (1) that hypoxia induces AngII expression and ROS generation, causing HCASMC proliferation and migration; (2) that the JNK pathway specifically mediates hypoxia-induced leptin expression in HCASMCs; (3) that hypoxia promotes AngII secretion and ROS generation that, in turn, affects leptin expression and genetic transcription in HCASMCs; and (4), that the relationship between AngII, ROS, leptin, and AP-1 (not HIF-1α) expression in HCASMCs under hypoxia results in artherogenesis.

The present study yielded similar results to a previous report that also examined leptin expression in HCASMCs subjected to hypoxia [23]. Under 2.5% O_2_ hypoxia, significant oxidative stress induced hypoxic signaling (JNK) and activated leptin expression. Our study identified the JNK pathway as the mediator of the hypoxia-induced leptin expression in HCASMCs. The JNK pathway has been reported to mediate the oxidative stress induced by leptin [24]. Moreover, hypoxia has been shown to stimulate JNK activation in a variety of cell types including HCASMCs. JNK mediates signals in response to cytokines and environmental stress, including oxidative stress. Previous studies indicated that JNK activation and ROS generation are immediate responses to hypoxia. Because the JNK pathway has been shown to be responsible for the hypoxic signaling, we hypothesized that either JNK1 siRNA or an ROS scavenger (NAC) could effectively attenuate hypoxia-induced JNK pathway activation.

The phosphorylation of JNK activates the JNK pathway. In previous studies, increases in JNK tyrosine phosphorylation occurred at an early hypoxic exposure time. In terms of the downstream effect of JNK activation, it is well documented that hypoxia leads to the activation of the AP-1 transcriptional factor that participates in cell response to hypoxia. Similarly, JNK phosphorylation enhanced oxidative phosphorylation and signal transduction to facilitate leptin expression; contrariwise, the JNK inhibitor, JNK siRNA, and NAC repressed the expression of leptin.

The relationship among AngII, ROS, and leptin, as well as their regulatory effects on genetic transcription in HCASMCs under hypoxia, has not yet been fully defined. AngII has been shown to induce HCASMC proliferation and migration under hypoxia. Hypoxia may regulate the expression of leptin in HCASMCs, and AngII may serve as an upstream mediator of leptin. Our study found that hypoxia-induced AngII secretion stimulated leptin expression, genetic transcription, and artherogenesis in HCASMCs, which could be effectively inhibited by ARB. Figure 3A showed that 2.5% hypoxia rapidly induced an increase in the levels of AngII within 1 h. So hypoxia may directly induce AngII release or protein synthesis, and indirectly mediated through cleavage of pro-AngII via activation of some enzymes. Previous study indicated that leptin requires AngII and Endithelin-1 (ET-1) co-stimulation for induction of rat VSMC hypertrophy [44]. This is achieved by leptin-induced increases in angiotensinogen, AngII type 1 (AT1) receptor, prepro- ET-1, and ET_A_ gene expression in rat VSMCs.

Our data also showed that ROS are involved in leptin-induced artherogenesis. Heightened ROS generation is closely associated with hypoxia. Studies have reported that ROS can modulate signaling pathways known to be involved in HCASMC proliferation and migration, including the JNK pathway. Increased production of ROS, furthermore, promotes HCASMC growth. ROS may be an upper mediator and a second messenger mediating intracellular signaling pathways. NAC effectively suppressed hypoxia-induced leptin expression, leptin and AP-1 binding, leptin-related genetic transcription in HCASMCs, and artherogenesis.

In different cell types, different MAPK pathways may be involved in the genetic transcription to leptin. Our study showed that AP-1, a downstream target of JNK, may play a role in the transcriptional regulation of leptin. The gel shift assay showed that leptin-DNA binding activity increased after hypoxia. This effect ceased with the use of a leptin-mutant plasmid, SP600125, and ARB. In HCASMCs, leptin- related genetic transcription occurs through AP-1 binding to the leptin promoter. In fact, the effects of hypoxia on the leptin promoter activity were eliminated when the AP-1 binding site was mutated.

Our study also demonstrated that hypoxia increased the presence of leptin in the nuclei of HCASMCs. This effect was effectively inhibited by NAC, JNK inhibitor, and leptin siRNA, indicating a relationship between AngII, ROS, the JNK pathway, and leptin. In addition, leptin, Dp44mT, or AngII, added exogenously, also increased the presence of leptin in the nuclei of HCASMCs.

## Conclusion

In conclusion, hypoxia in cultured HCASMCs induces the expression of AngII and ROS. Ang II, ROS, and the JNK pathway mediate the induction of leptin expression with subsequent artherogenesis through increased transcriptional activity in HCASMCs. Artherogenesis in HCASMCs under hypoxia can be identified by increased proliferation and migration activity. Hypoxia-induced AngII expression has similar effects to that of exogenously added AngII and may increase both leptin expression and transcriptional activity in HCASMCs.

## Additional files

Additional file 1: Figure S1. Effect of different degree of hypoxia on the expression of leptin protein in HCASMCs. (A and B) Leptin protein expression was measured in HCASMCs subjected to different degree of hypoxia (10%, 5%, and 2.5% O2) for 4 h. Leptin expression was notably increased by 2.5% hypoxia for 4 h in comparison to the other hypoxia conditions. ^*^P < 0.01 *vs.* normoxia control (n = 3). Additional file 2: Figure S2. Inhibitory effect of JNK1 siRNA on the phosphorylation of JNK under hypoxia in HCASMCs. (A and B) JNK1 siRNA inhibited 60% of JNK phosphorylation compared with control siRNA after 2.5% hypoxia for 2 h. ^#^P < 0.01 *vs.* control siRNA (n = 3). Additional file 3: Figure S3. Hypoxia-induced leptin protein expression is not inhibited by HIF-1α siRNA under hypoxia in HCASMCs. (A and B) Hypoxia with 2.5% hypoxia increased leptin protein expression in HCASMCs. HIF-1α siRNA could not inhibit hypoxia-induced leptin protein expression. *P < 0.01 *vs.*normoxia control. ^#^P < 0.01 *vs.* hypoxia (n = 3). Additional file 4: Figure S4. Hypoxia increases the presence of leptin in the nuclei of HCASMCs. Hypoxia increased the presence of leptin (green color) in the nuclei (purple color) of HCASMCs (red color), which could by inhibited by SP600125, losartan, NAC, and leptin siRNA. In addition, exogenously addition of AngII, Dp44mT, or leptin under normoxia also increased the presence of leptin in the nuclei of HCASMCs (n = 6).

## Abbreviations

CVDs: Cardiovascular diseases
VSMC: Vascular smooth muscle cell
HCASMC: Human coronary arterial smooth muscle cell
ROS: Reactive oxygen species
AngII: Angiotensin II
JNK: c-Jun N-terminal kinase
mAbs: Monoclonal antibodies
MAPK: Mitogen-activated protein kinase
ERK: Extracellular signal-regulated kinase
ARBs: AngII type 1 receptor blockers
NAC: *N*-Acetyl-l-cysteine
HIF-1α: Hypoxia-inducible factor-1α
siRNA: Small interfering RNA
ELISA: Enzyme-linked immunosorbent assay
EMSA: Electrophoretic mobility shift assay
AP-1: Activator protein 1
VEGF: Vascular endothelial growth factor
NO: Nitric oxide
ET-1: Endothelin-1
