# Supplementary figures and images for: Molecular regulation of the expression of leptin by hypoxia in human coronary artery smooth muscle cells
Source: J Biomed Sci. 2015 Jan 9;22(1):5. doi: 10.1186/s12929-014-0109-8 (PMC4298872; doi:10.1186/s12929-014-0109-8)

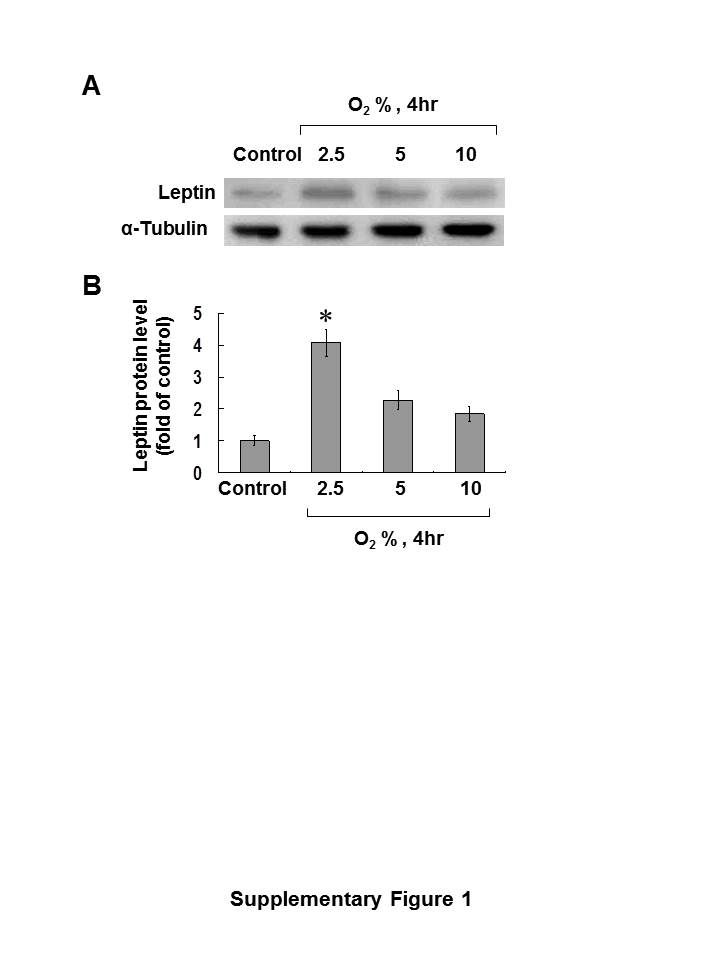

Supplement: Additional file 1: Figure S1. — Effect of different degree of hypoxia on the expression of leptin protein in HCASMCs. (A and B) Leptin protein expression was measured in HCASMCs subjected to different degree of hypoxia (10%, 5%, and 2.5% O2) for 4 h. Leptin expression was notably increased by 2.5% hypoxia for 4 h in comparison to the other hypoxia conditions. *P < 0.01 vs. normoxia control (n = 3). [file 12929_2014_109_MOESM1_ESM.jpeg]

## Slide 1
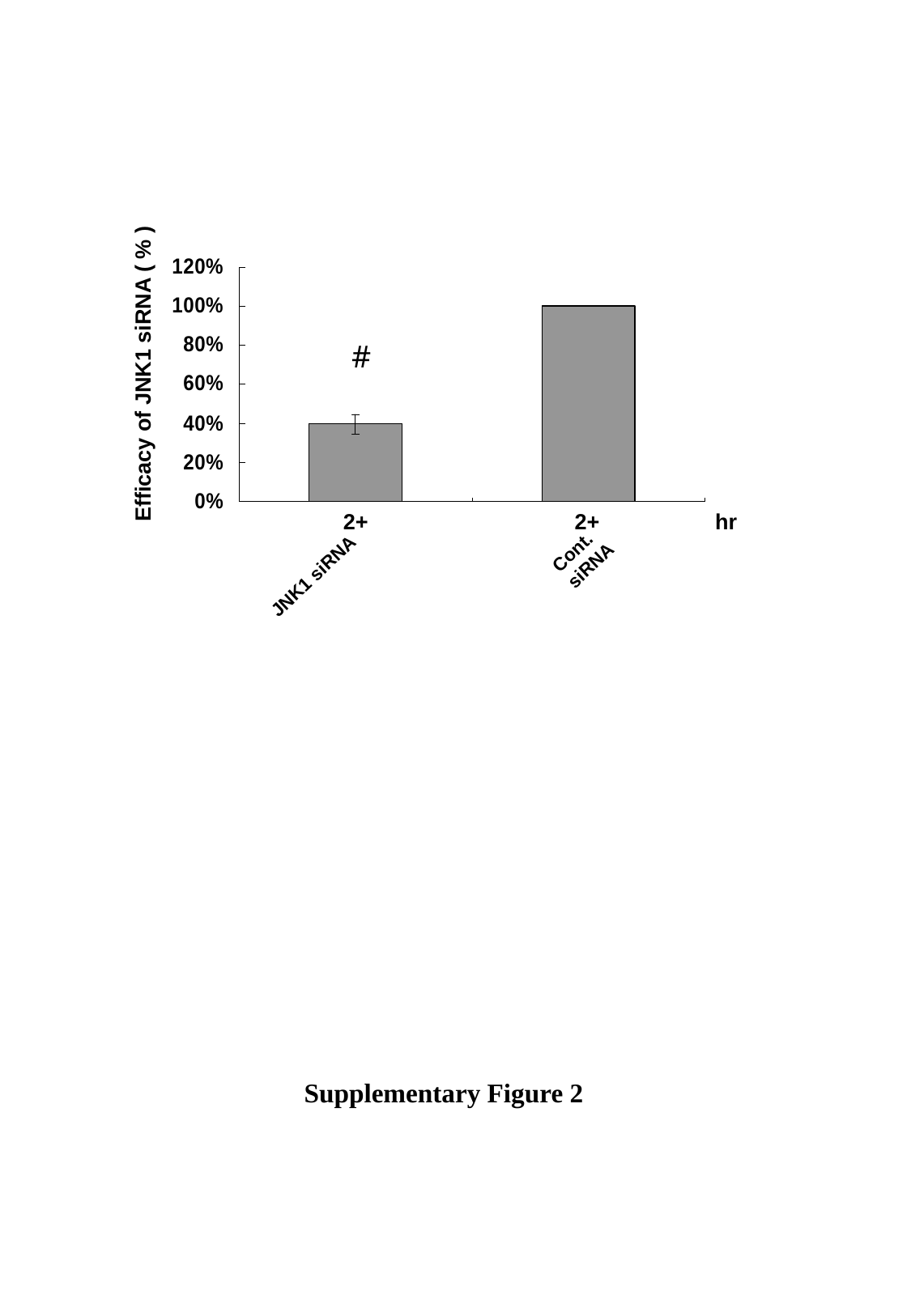

#
Efficacy of JNK1 siRNA ( % )
2+ 2+ hr
Cont. siRNA
JNK1 siRNA
Supplementary Figure 2

Supplement: Additional file 2: Figure S2. — Inhibitory effect of JNK1 siRNA on the phosphorylation of JNK under hypoxia in HCASMCs. (A and B) JNK1 siRNA inhibited 60% of JNK phosphorylation compared with control siRNA after 2.5% hypoxia for 2 h. #P < 0.01 vs. control siRNA (n = 3). [file 12929_2014_109_MOESM2_ESM.ppt]

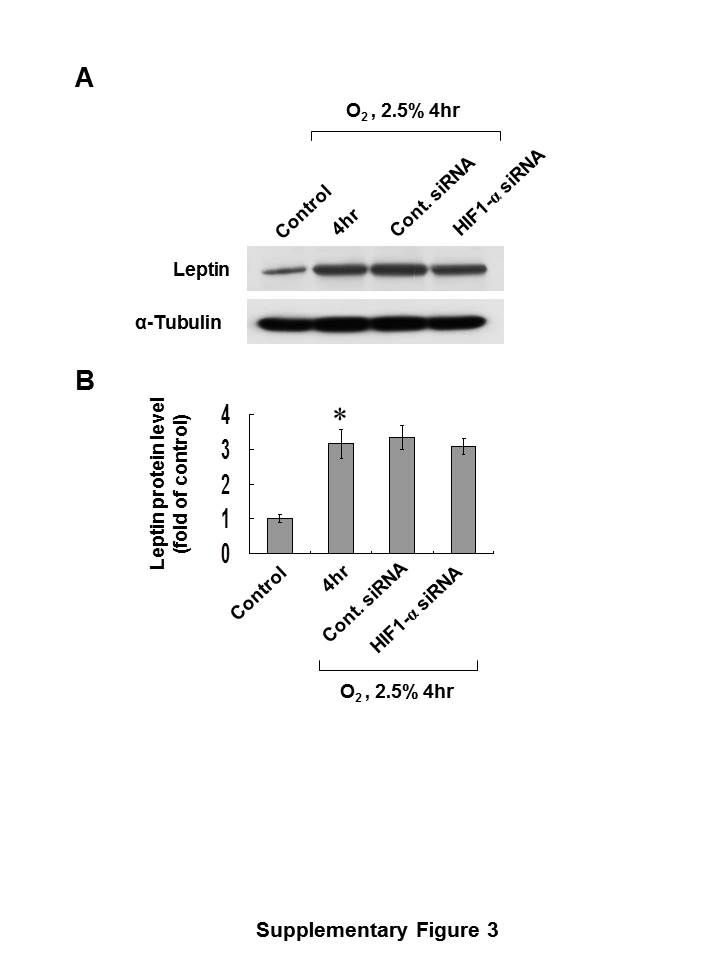

Supplement: Additional file 3: Figure S3. — Hypoxia-induced leptin protein expression is not inhibited by HIF-1α siRNA under hypoxia in HCASMCs. (A and B) Hypoxia with 2.5% hypoxia increased leptin protein expression in HCASMCs. HIF-1α siRNA could not inhibit hypoxia-induced leptin protein expression. *P < 0.01 vs.normoxia control. #P < 0.01 vs. hypoxia (n = 3). [file 12929_2014_109_MOESM3_ESM.jpeg]

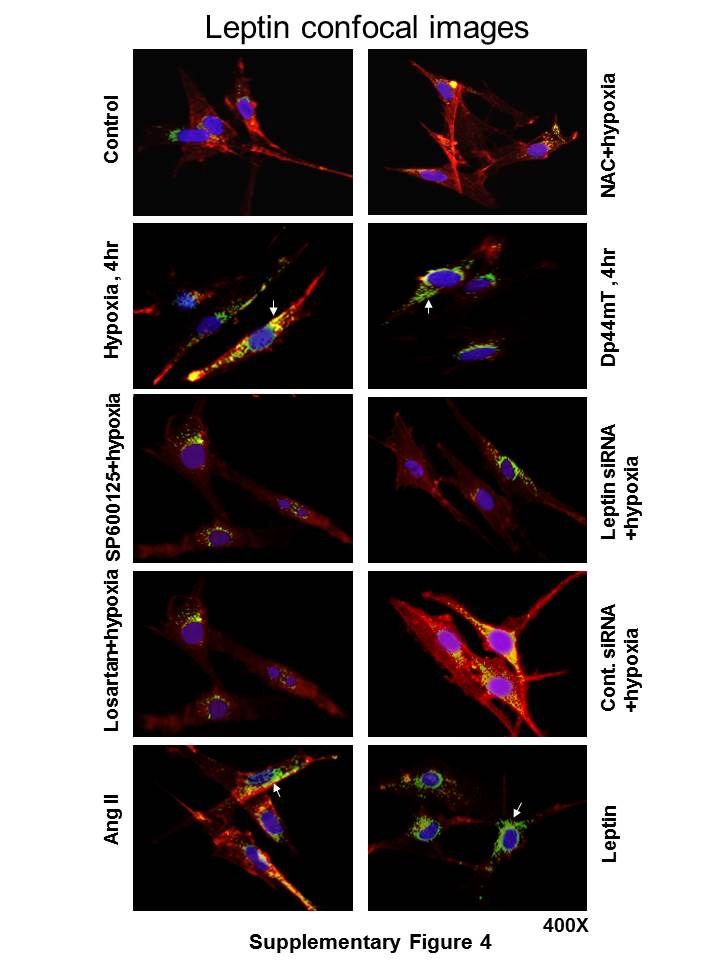

Supplement: Additional file 4: Figure S4. — Hypoxia increases the presence of leptin in the nuclei of HCASMCs. Hypoxia increased the presence of leptin (green color) in the nuclei (purple color) of HCASMCs (red color), which could by inhibited by SP600125, losartan, NAC, and leptin siRNA. In addition, exogenously addition of AngII, Dp44mT, or leptin under normoxia also increased the presence of leptin in the nuclei of HCASMCs (n = 6). [file 12929_2014_109_MOESM4_ESM.jpeg]
